# Photoreduced Amino Acid–Capped AgCu Nanohybrids: Precision Tools for Seed‐Borne Pathogen Eradication With Concurrent Growth Promotion in Sunflower Systems

**DOI:** 10.1155/ijfo/9943535

**Published:** 2025-10-06

**Authors:** Isabela Santos Lopes, Liudmila Trotsiuk, Théo Duarte, Régis Deturche, Léa Le Joncour, Safi Jradi, Bianca Natasha Oliveira de Moraes, Susana de Souza Barreto, Marcia Regina Franzolin, Christophe Couteau, Lilia Coronato Courrol, Muhammad Asif Zahoor

**Affiliations:** ^1^ Institute of Environmental, Chemical, and Pharmaceutical Sciences (ICAQF-UNIFESP), Federal University of São Paulo (UNIFESP), Diadema, Brazil, unifesp.br; ^2^ Light, Nanomaterials and Nanotechnologies-L2n, University of Technology of Troyes-UTT & CNRS UMR 7076, Troyes, France; ^3^ Laboratory of Mechanical & Material Engineering-LASMIS, University of Technology of Troyes-UTT, Troyes, France; ^4^ Bacteriology Laboratory, Butantan Institute, São Paulo, Brazil, butantan.gov.br

**Keywords:** antimicrobial, gamma-aminobutyric acid, methyl aminolevulinate, seed nanopriming, silver–copper nanoparticles, sunflower

## Abstract

Seed‐borne pathogens significantly threaten crop health and food safety, negatively affecting plant growth and triggering public health risks. Traditional seed treatments with chemical fungicides have limitations, including environmental toxicity and pathogen resistance. Seed nanopriming, an advanced nanobiotechnology approach, offers a sustainable alternative. This research introduces two innovative copper–silver hybrid nanoparticles for sunflower seed nanopriming applications. The nanoparticles were synthesized via a photoreduction approach using methyl aminolevulinate (MALA) and gamma‐aminobutyric acid (GABA) as dual‐function agents, biocompatible stabilizers, and growth enhancers. These amino acid derivatives were selected for their established roles in plant stress response and unique photodynamic properties. Structural characterization revealed crystalline AgCu composites with AgCl phases, displaying spherical morphology with narrow size distributions (22 and 31 nm diameter) and exceptional colloidal stability. Antimicrobial testing showed remarkable efficacy, with 10% nanoparticle solutions achieving > 84% inhibition of *Staphylococcus aureus* while eliminating *Escherichia coli* and *Candida albicans* populations. In seed priming trials, GABA‐functionalized nanoparticles (GABAAgCu) demonstrated superior performance, increasing seed vigor by 133% and significantly boosting antioxidant defenses compared to controls. This treatment consistently improved germination parameters and early seedling development. The MALA‐conjugated nanoparticles (MALAAgCu) exhibited a more complex interaction, enhancing seed vigor by 50% but inducing oxidative stress that compromised germination rates, potentially due to observed seed coat microstructural alterations. FLIM analysis indicated that both nanoparticle types enhanced chlorophyll fluorescence lifetimes, suggesting improved Photosystem II efficiency. These results highlight the potential of amino acid–tailored bimetallic nanoparticles as multifunctional agricultural tools, offering simultaneous pathogen control and physiological enhancement while addressing sustainability challenges in modern crop production.

## 1. Introduction

Sunflowers, *Helianthus annuus*, are economically significant crops cultivated globally for their oil‐rich seeds [1]. However, the cultivation of sunflowers is becoming increasingly vulnerable to the adverse effects of climate change and global warming [2]. The increasing frequency of extreme weather events, combined with rising temperatures and shifting precipitation regimes, poses a dual threat to global agriculture by simultaneously diminishing crop yields and impairing seed quality parameters, particularly concerning the given seeds′ pivotal role in developing climate‐resilient agricultural systems [3].

These climate‐driven challenges are further exacerbated by evolving seed‐borne pathogen dynamics. Fungi, bacteria, and viruses are adapting to new environmental conditions, employing multiple mechanisms to impair germination, including tissue destruction, phytotoxin release, and nutrient competition [4]. While conventional fungicides have historically controlled these pathogens, their declining efficacy due to environmental toxicity and resistance development necessitates alternative solutions [5].

Green nanotechnology integrates sustainable principles into nanoscale science, developing eco‐friendly nanomaterials and processes that minimize environmental harm while maximizing efficiency [6]. Employing biobased synthesis, renewable energy, and nontoxic methods enables the cleaner production of advanced solutions for renewable energy, pollution remediation, and precision medicine, all designed with complete lifecycle safety, ensuring biodegradability or recyclability. This innovative approach demonstrates how cutting‐edge technology can address global challenges while maintaining ecological integrity, offering a responsible pathway to technological progress that aligns with environmental preservation.

Green nanotechnology emerges as a promising approach to address these interconnected challenges. Nanoparticle (NP)‐based seed priming offers dual functionality: antimicrobial action against pathogens and physiological enhancement of seeds [7, 8]. This innovative strategy confers broad‐spectrum stress tolerance, equipping plants to withstand abiotic challenges (drought, salinity, and thermal extremes) and biotic threats (bacteria, viruses, fungi, and pests) [9–11].

By significantly improving seed germination and vigor, NPs can empower plants to withstand the challenges posed by climate change, including extreme weather events and rising temperatures [9, 12].

Building on this foundation, amino acid–based priming agents like aminolevulinic acid (ALA) have successfully enhanced stress tolerance through improved chlorophyll synthesis and photosynthetic efficiency [13]. Its derivative, methyl aminolevulinate (MALA), offers greater stability and bioavailability, enabling more efficient Protoporphyrin IX production for sustained plant growth promotion, though its seed priming potential remains unexplored [14, 15]. Similarly, *γ*‐aminobutyric acid (GABA) has emerged as an effective priming agent through reactive oxygen species (ROS) scavenging and stress tolerance enhancement [16, 17].

Notably, both MALA and GABA exhibit secondary benefits as photoactive pesticides, inducing Protoporphyrin IX accumulation in microorganisms for sunlight‐activated pathogen inactivation [18]. This multifunctionality aligns with the advantages of metallic NPs, particularly silver and copper formulations (AgNPs and CuNPs), which serve dual roles as growth promoters and antimicrobial agents [19, 20]. Bimetallic silver–copper nanoparticles (AgCuNPs) demonstrate superior antimicrobial activity against diverse pathogens compared to monometallic versions or commercial fungicides [21]. This synergistic effect enhances their antimicrobial efficacy across a wide range of pathogens, including bacteria, fungi, and viruses [22]. Recently, it was observed that AgCuNPs exhibit superior efficacy compared to commercially available fungicides against plant pathogens, *Alternaria grandis* and *Fusarium oxysporum* [23].

This study employs green synthesis methods to develop novel NPs using MALA/GABA to produce AgCuNPs via photoreduction [24–26]. After comprehensive physicochemical characterization, we evaluated antimicrobial activity against model pathogens (*Staphylococcus aureus*, *Escherichia coli*, and *Candida albicans*) and applied the NPs as sunflower seed primers. Treatment efficacy was assessed through multiple parameters: hydrogen peroxide (H_2_O_2_) levels, oxidative stress markers, germination percentage (GP), vigor index (Vi), chlorophyll content, and chlorophyll fluorescence lifetime via fluorescence lifetime imaging microscopy (FLIM), providing a multifaceted evaluation of photosynthetic efficiency and plant development.

## 2. Materials and Methods

### 2.1. Materials

All chemicals were acquired from certified suppliers: Methyl *δ*‐aminolevulinate hydrochloride (A5575, ≥ 98% purity) and GABA (A2129, ≥ 99% purity) from Sigma‐Aldrich (United States) and cupric chloride dihydrate (CuCl_2_·2H_2_O, 99% purity) from Êxodo Científica (Brazil). Silver nitrate (AgNO_3_) and polyethylene glycol (PEG) 10000 were obtained from Sigma‐Aldrich (United States). All solutions were prepared using ultrapure water.

### 2.2. NP Synthesis

Monometallic AgNPs and CuNPs, as well as bimetallic AgCuNPs, were synthesized via photoreduction using aqueous solutions of AgNO_3_ (1 mM) and/or copper chloride (10 mM), with PEG (0.1 mM) as a stabilizer. For functionalization, NPs were modified with either 8 mM MALA (MALANPs) or 22 mM GABA (GABANPs). Under a 300‐W xenon lamp (3.6 W/cm^2^ intensity at 10 cm distance), the optimal irradiation durations were 4 min for AgNPs, 7 min for CuNPs, and 5 min for AgCuNPs.

### 2.3. Physicochemical Characterization

#### 2.3.1. Spectroscopic Analysis

UV–Vis absorption spectra were recorded using a JASCO V‐730 spectrophotometer (Jasco, Japan) with 1 cm pathlength quartz cuvettes.

FTIR spectra (4000–400 cm^−1^) were acquired on a PerkinElmer Spectrum Two spectrometer (United States) with 4 cm^−1^ resolution (32 scans).

#### 2.3.2. Electron Microscopy

NP morphology was characterized using field‐emission scanning electron microscopy (FE‐SEM, Hitachi SU8030, 5 kV) and transmission electron microscopy (JEOL JEM 2100), with samples deposited on substrates and carbon‐coated copper grids, respectively.

#### 2.3.3. Colloidal Properties

Dynamic light scattering determined the zeta potential and polydispersity index (Malvern Zetasizer Nano ZS, United Kingdom). Reported values represent triplicate measurements.

#### 2.3.4. Crystallographic Analysis

X‐ray diffraction patterns (10°–90° 2*θ* range, 0.05° step size) were collected using a Bruker D8 Discover diffractometer with Cu K*α* radiation (*λ* = 1.5418 Å, 40 kV, 35 mA). A Pilatus 2D detector recorded diffracted intensities integrated over ≈25°.

### 2.4. Antimicrobial Susceptibility Testing

The antimicrobial efficacy of synthesized NPs (GABAAg, GABACu, GABAAgCu, and MALAAgCu) was evaluated against reference microbial strains: *E. coli* ATCC 25922, *S. aureus* ATCC 25923, and *C. albicans* ATCC 10231, following standardized Clinical and Laboratory Standards Institute (CLSI) protocols.

#### 2.4.1. Assay Preparation

Microbial suspensions were prepared in appropriate media: bacteria, Mueller–Hinton (MH) broth, and yeast, Sabouraud (Sab) broth. The final inoculum concentration was standardized to 10^6^ CFU/mL.

#### 2.4.2. Experimental Setup

Test wells received 50 *μ*L microbial suspension (10^6^ CFU/mL) and 50 *μ*L test solution (either sterile medium control or 10% NP solution in respective broth).

Final well characteristics are as follows: total volume: 100 *μ*L, microbial load: 10^4^ CFU/well, and NP concentration: 5% (v/v).

#### 2.4.3. Incubation and Analysis

Microplates were incubated at 37°C for 20 h. Microbial growth was quantified by measuring optical density at 595 nm (Multiskan EX plate reader, Thermo Scientific). Growth inhibition was calculated using the following formula:

Inhibition %=1−OD595treatedOD595control×100.

Results were expressed as mean ± standard deviation of triplicate measurements.

Gentamicin was used at 2 *μ*g/mL and Amphotericin B at 1 *μ*g/mL as positive controls for bacteria and yeast, respectively. Untreated microorganisms were used as the negative control.

### 2.5. Seed Preparation

Sunflower seeds (*Helianthus annuus* L.) obtained from Germiverde Sementes (Brazil) were used in this study. Ninety viable seeds were randomly selected and primed for 24 h at 25^°^C ± 4^°^C using three treatments: (i) water control, (ii) 10% MALAAgCu NPs, and (iii) 10% GABAAgCu NPs (5 mL per treatment).

### 2.6. *In Vitro* Seed Germination

After NP treatment, all seeds were carefully washed to remove surface residues and categorized for subsequent analyses. Two complementary experiments were conducted to evaluate different aspects of seed performance.

#### 2.6.1. Experiment 1: Germination Parameters

A sample size of *n* = 20 seeds per priming group was used for this experiment. The seeds were cultured in angled containers (45°) with adequate ventilation to ensure optimal conditions. The experiment lasted for 10 days, with daily hydration to maintain moisture levels. The primary endpoint assessed was the GP, which was recorded to determine the effectiveness of the NP treatment on seed viability and sprouting success.

#### 2.6.2. Experiment 2: Early Growth Characteristics

This experiment utilized a smaller sample size of *n* = 10 seeds per group, planted in standard potting soil at a depth of 2 mm. The seeds were maintained with daily watering to support growth. Over a 12‐day observation period, key developmental parameters were measured, including hypocotyl elongation and leaf emergence. These metrics provided insights into the early growth dynamics and potential physiological effects of the NP priming on seedling establishment.

Both experiments were designed to provide a comprehensive understanding of the NP treatment′s impact on seed germination and subsequent early stage plant development.

### 2.7. Effect of H_2_O_2_ on NPs

Following the manufacturer′s instructions, the concentration of H_2_O_2_ in the priming solutions was determined using a commercial colorimetric detection kit (Elabscience Biotechnology Inc., United States). Absorbance measurements were performed using a spectrophotometric method at the recommended wavelength.

### 2.8. Measurement of Physiological Indices

Germination was defined as visible radicle protrusion through the seed coat, and quantitative assessments included GP, calculated as (germinated seeds/total seeds) × 100, along with morphometric parameters such as root length (RL) and shoot length (SL). Additionally, the Vi was determined using the formula (RL + SL) × GP. Measurements were recorded at 3, 6, and 10 days postgermination, with eight biological replicates per treatment group, and results were expressed as mean ± standard deviation.

### 2.9. Oxidative Stress Markers

For enzymatic analysis, NP‐treated seedlings (*n* = 10 biological replicates) from Experiment 1 were cryogenically homogenized following liquid nitrogen immersion. Precisely weighed samples (0.108 ± 0.003 g fresh weight) were homogenized in 5 mL phosphate‐buffered saline (PBS, pH 7.4) and centrifuged (4000 × g, 15 min, 4°C). The resultant supernatant was aliquoted for total superoxide dismutase (T‐SOD) activity determination and reduced glutathione (GSH) quantification using validated commercial assay kits (Elabscience Biotechnology). After manufacturer‐specified protocols, measurements were performed at two postpriming intervals (6 and 10 days).

### 2.10. Chlorophyll Fluorescence and Content

Chlorophyll fluorescence characteristics were assessed in primary leaves (12‐day‐old seedlings from Experiment 2) using a Fluorolog 3 spectrofluorometer (Horiba Scientific) with 435 nm excitation wavelength delivered via a fiber optic probe. Parallel chlorophyll quantification was performed using a portable chlorophyll meter (Falker), which employs multispectral analysis to calculate the Falker Chlorophyll Index (FCI)—a unitless value positively correlated with chlorophyll concentration. Six replicate measurements per treatment group were averaged for statistical analysis.

### 2.11. FLIM Analysis

Chlorophyll fluorescence lifetime analysis was conducted using a confocal time‐correlated single photon counting (TCSPC) system (DCS‐120, Becker & Hickl) coupled to a ZEISS LSM 980 microscope. Cotyledon samples from Experiment 1 were imaged at 6 and 10 days posttreatment. The excitation source was a 473 nm picosecond pulsed laser (80 MHz repetition rate) with emission collected through a 520‐nm long‐pass filter. Imaging parameters included 20× objective (NA 0.8), 180 s acquisition time, and manual ROI selection based on chlorophyll fluorescence.

Fluorescence decay kinetics were modeled using

(1) I=A1e−t/τ1+A2e−t/τ2

where *τ*ᵢ represents component lifetimes and *A*ᵢ their amplitudes. Fit quality was verified through *χ* ^2^ analysis and residual distribution. The amplitude‐weighted mean lifetime was calculated as

(2) τm=∑biτi2∑biτi.

### 2.12. Statistical Analysis

Statistical analyses were conducted using OriginPro 8.5 (OriginLab Corporation). A two‐factor analysis of variance (ANOVA) was employed to evaluate treatment effects, with a significance threshold level of *p* < 0.05. Descriptive statistics (*m* *e* *a* *n* ± *s* *t* *a* *n* *d* *a* *r* *d* *d* *e* *v* *i* *a* *t* *i* *o* *n*) were computed for all experimental groups and their respective controls.

## 3. Results

### 3.1. NP Characterization

#### 3.1.1. UV–Visible Spectroscopy

The UV–Vis absorption profiles of the synthesized NPs are displayed in Figure 1a. MALAAgCu and GABAAgCu show characteristic peaks at approximately 275 nm, consistent with AgCl NP formation [27]. The spectra exhibit broad absorption across the visible range (300–800 nm), which we attribute to overlapping surface plasmon resonance bands from metallic silver (390–420 nm) and copper (560–600 nm) NPs, as evidenced by monometallic samples (GABAAg and GABACu) and supported by literature reports [28, 29].

Figure 1(a) UV–Vis and (b) FTIR spectra of MALA, GABA, GABAAg, GABACu, GABAAgCu, and MALAAgCu.

**Figure:**
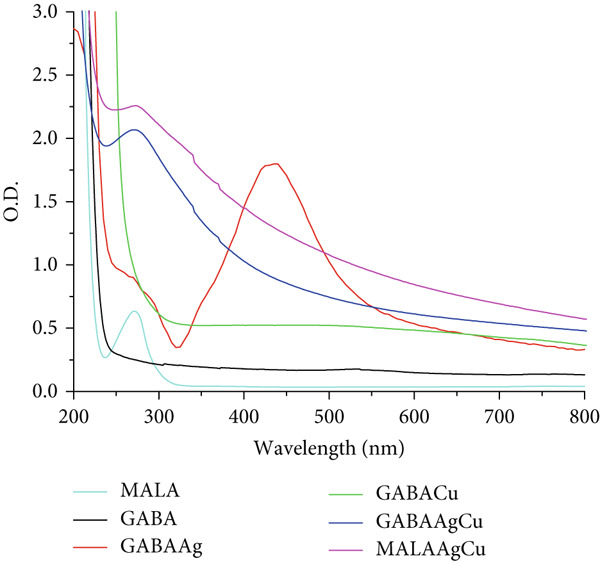
(a)

**Figure:**
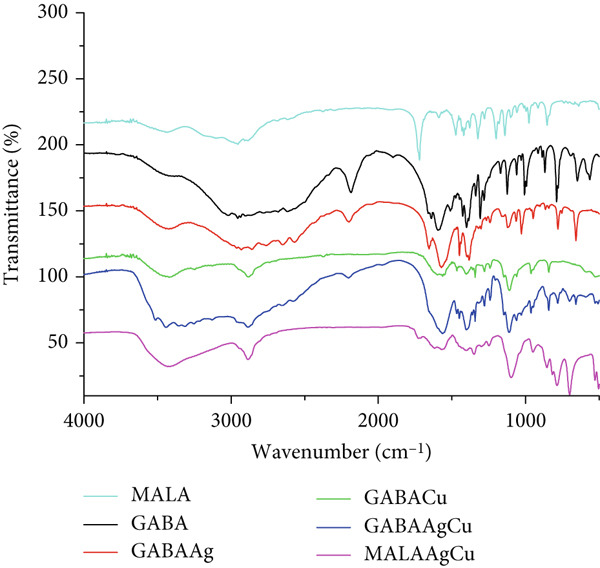
(b)

#### 3.1.2. FTIR Spectroscopy

The FTIR spectra in Figure 1b reveal characteristic vibrational modes for MALA, GABA, and their corresponding NPs. Key absorption bands include 3440 cm^−1^ (O‐H stretch), 3028 cm^−1^ (NH_3_^+^ asymmetric stretch), 2874 cm^−1^ (CH_2_ asymmetric stretch), 1653 cm^−1^ (NH_3_ deformation), 1465 cm^−1^ (CH_2_ scissoring), 1403 cm^−1^ (NH_3_ rocking), and 999 cm^−1^ (COO^-^ wagging) [30]. Notably, enhanced absorption between 1200 and 990 cm^−1^ indicates strong NP–amino acid interactions, primarily through the coordination of amino groups (‐NH_2_) with Ag/Cu metal centers. These spectral modifications arise from perturbations in C‐N stretching and bending vibrations upon metal complexation.

#### 3.1.3. Surface Charge and Size of the NPs

The NPs demonstrated exceptional colloidal stability, maintaining their physicochemical properties for > 180 days. Surface charge analysis revealed negative zeta potentials of −14.3 mV (MALAAgCu) and −23.2 mV (GABAAgCu), indicating electrostatic stabilization. Size distribution analysis showed superior monodispersity for GABAAgCu (photodynamic inactivation [PDI] = 0.189) compared to MALAAgCu (PDI = 0.248), suggesting more homogeneous NP formation.

#### 3.1.4. TEM

**Figure 2:**
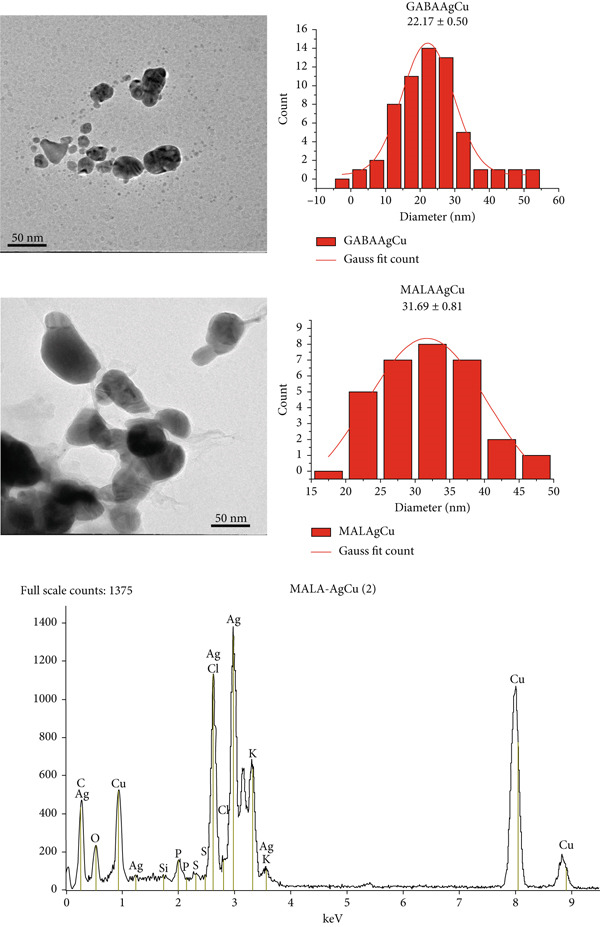
TEM images and size distribution of GABAAgCu and MALAAgCu, including EDS study.

Transmission electron microscopy analysis (Figure 2) reveals that both GABAAgCu and MALAAgCu NPs possess spherical geometries. Quantitative size measurements show a distinct difference, with GABAAgCu particles averaging 22 nm in diameter compared to 32 nm for MALAAgCu NPs, representing a 31% reduction in particle size.

The EDS spectrum of MALAAgCu reveals the elemental composition of the bimetallic NPs. Dominant peaks at ~2.98 (Ag L*α*) and ~3.15 keV (Ag L*β*) confirm the presence of Ag. Clear peaks at ~8.04 (Cu K*α*) and ~8.90 keV (Cu K*β*) verify Cu incorporation. Lower intensity suggests less Cu than Ag, consistent with the reported 2.7% w/w Ag. Peak at ~2.62 keV (Cl K*α*) may indicate residual salts (e.g., AgCl).

#### 3.1.5. XRD Pattern

**Figure 3:**
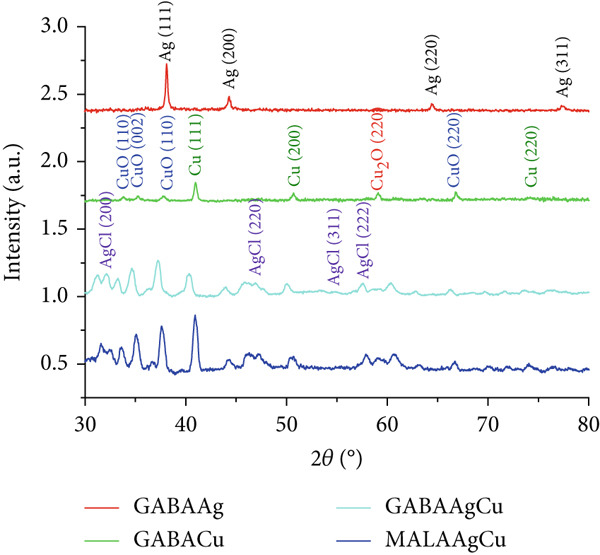
XRD patterns of GABAAg, GABACu, GABAAgCu, and MALAAgCu.

Figure 3 presents the XRD patterns of GAGAAg, GABACu, GABAAgCu, and MALAAAgCu. Diffraction peaks at 37.9°, 44.3°, 64.4°, and 77.4° in GABAAg correspond to the (111), (200), (220), and (311) planes of the face‐centered cubic (fcc) silver structure (JCPDS No. 04‐0783) [31, 32]. The presence of copper nanostructures was confirmed by the observation of distinct XRD peaks at 40.6°, 50.1°, 74.1°, and 90.4°, corresponding to the (111), (200), (220), and (311) planes of fcc copper. These peaks align with the standard data for metallic copper (JCPDS No. 04‐0836) [33]. Additional peaks in GABACu NPs indicated the presence of CuO and Cu_2_O (JCPDS Nos. 01‐080‐0076 and 01‐077‐0199). Diffraction peaks at 32.2°, 46.2°, 54.8°, and 57.5° were attributed to the (111), (200), (220), and (311) planes of AgCl NPs (JCPDS Card No. 31‐1238) [34]. In the case of AgCuNPs, a shift and broadening of peaks corresponding to the (111) planes of fcc Cu and fcc Ag suggested the formation of copper–silver alloys.

The Debye–Scherrer equation (Equation (3)) was used to estimate the average crystallite size in the synthesized AgCuNPs:

(3) D=0.94 λβ cos θ 

where *D* is the average crystallite size (nanometer), *λ* is the x‐ray wavelength (0.15418 nm), *θ* is the Bragg angle of the diffraction peak, and *β* is the full width at half maximum (FWHM) of the diffraction peak. Two silver and copper peaks were used to calculate average crystallite sizes. The obtained values for silver were 14.05 and 16.46 nm for GABAAgCu and MALAAgCu, respectively. For copper, the values found were 14.69 and 14.26 nm for MALAAgCu and GABAAgCu, respectively.

### 3.2. Antimicrobial Activity

**Figure 4:**
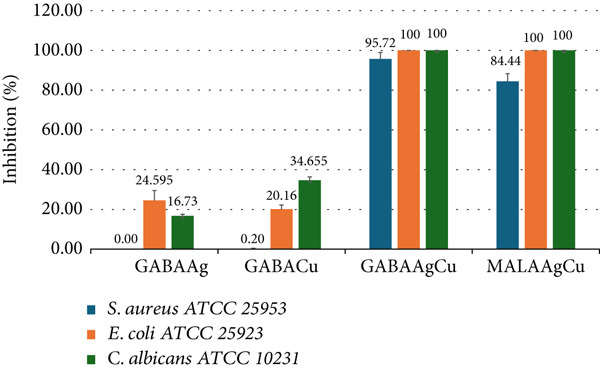
The antimicrobial activity of GABAAg, GABACu, GABAAgCu, and MALAAgCu nanoparticles against *S. aureus* ATCC 25923, *E. coli* ATCC 25923, and *C. albicans* ATCC 10231.

Figure 4 illustrates the antimicrobial activity of mono‐ and bimetallic NPs against *E. coli*, *S. aureus*, and *C. albicans*. Comparative antimicrobial assessment revealed significant differences between NPs containing equal molar ratios of Ag (1 mM) and Cu (10 mM) when tested at 5% dilution. While GABAAg and GABACu exhibited lower inhibition rates (< 35%), GABAAgCu exhibited broad‐spectrum antimicrobial activity, inhibiting ~96% of *S. aureus* and eliminating *E. coli* and *C. albicans*.

MALAAgCu NPs displayed similar antimicrobial activity, inhibiting ~84% of *S. aureus* and 100% of *E. coli* and *C. albicans*.

Our findings show that GABAAgCu and MALAAgCu NPs achieve 100% inactivation of *C. albicans*, suggesting their possible efficacy against plant‐pathogenic fungi. These results align with and extend previous reports demonstrating the superior antimicrobial efficacy of silver–copper bimetallic systems [35–37].

### 3.3. Seed Nanopriming

#### 3.3.1. Priming Solution

Figure 5a,b shows the UV–visible and FTIR spectra of the postpriming solutions after seed removal.

Figure 5(a) UV–visible and (b) FTIR spectra of MALAAgCu and GABAAgCu priming solutions after seed removal.

**Figure:**
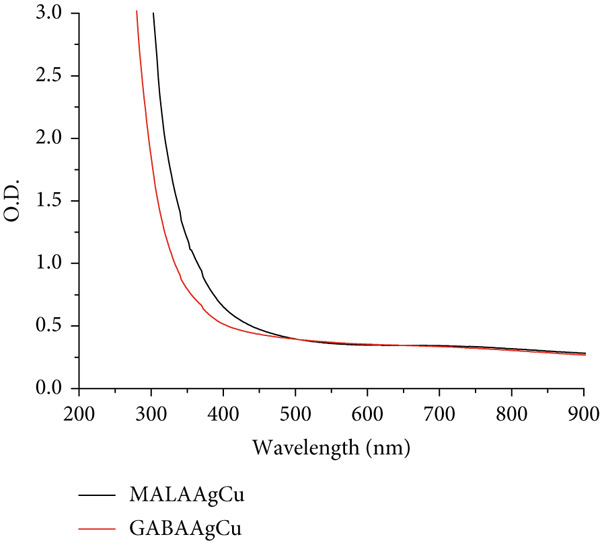
(a)

**Figure:**
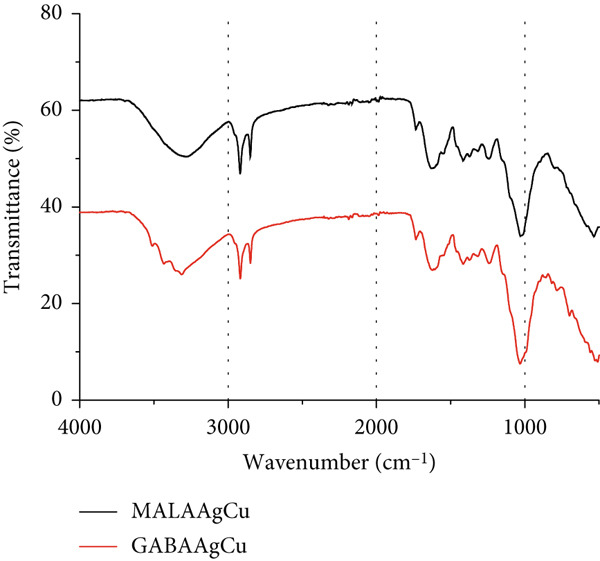
(b)

UV–visible analysis (Figure 5a) revealed a decrease in visible band intensity and an increase in UV absorption bands, suggesting the successful incorporation of NPs into the seeds and the potential release of specific seed components into the solution.

Following nanopriming (Figure 5b), priming solutions exhibited a peak between 1000 and 1250 cm^−1^, attributed to C‐O bond stretching vibrations, indicating the presence of organic acids, phenolic compounds, and carotenoids.

#### 3.3.2. Seed Coats

**Figure 6:**
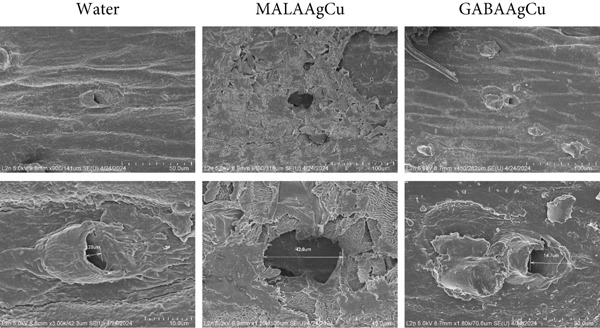
SEM images of seed coats obtained after priming treatments (superior line with scale of 50 *μ*m and inferior line with scale of 10 *μ*m).

The detached seed coats were examined using scanning electron microscopy (SEM) illustrated in Figure 6. The SEM data demonstrate that priming treatments physically and chemically modify seed coat architecture. Coats of seeds treated with water exhibited pores of approximately 3.3 *μ*m. MALAAgCu‐treated seed coats had significantly larger pores, measuring around 42.8 *μ*m. GABAAgCu‐treated seed coats had pores of ~14.7 *μ*m with a roughened surface with cracks.

#### 3.3.3. H_2_O_2_ Content in Priming Solutions

Figure 7a presents the H_2_O_2_ content in the postpriming NP solution analysis. As observed, NP solutions present significantly higher H_2_O_2_ levels than water solutions. The relative increases in H_2_O_2_ content were approximately 5.7‐ and 3.6‐fold for MALAAgCu and GABAAgCu, respectively.

Figure 7Impacts of MALAAgCu and GABAAgCu in (a) H_2_O_2_ content postpriming solutions and (b) vigor index.

**Figure:**
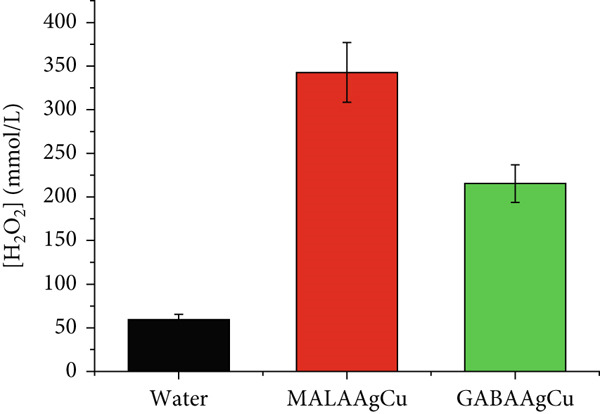
(a)

**Figure:**
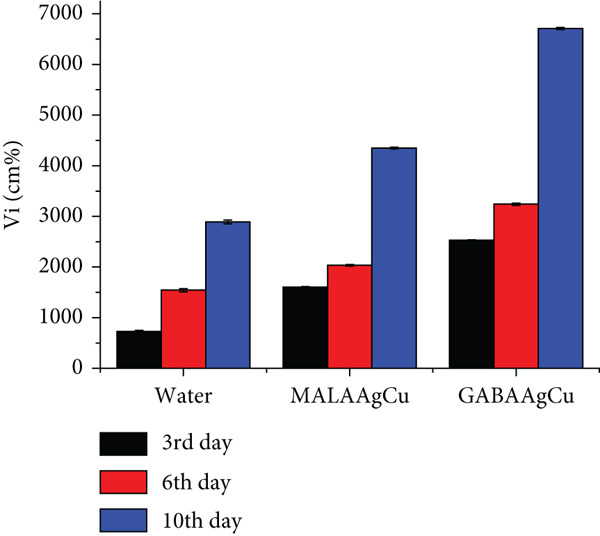
(b)

#### 3.3.4. Germination Rate and Vi

Germination started on the 2nd or 3rd day after priming. The GPs of seeds subjected to nanopriming were 80% and 96.7% for MALAAgCu and GABAAgCu, respectively, against 66.7% for the water group.

The Vi was monitored throughout the experiment, with results shown in Figure 7b. NP treatments enhanced the Vi on Days 3, 6, and 10. GABAAgCu demonstrated the most significant germination increase compared to the control during the experiment. On the 10th day, GABAAgCu treatment led to a 2.3‐fold increase in Vi, while MALAAgCu treatment resulted in a 1.6‐fold increase. This indicates that seedlings from MALAAgCu‐treated seeds grew more slowly.

**Figure 8:**
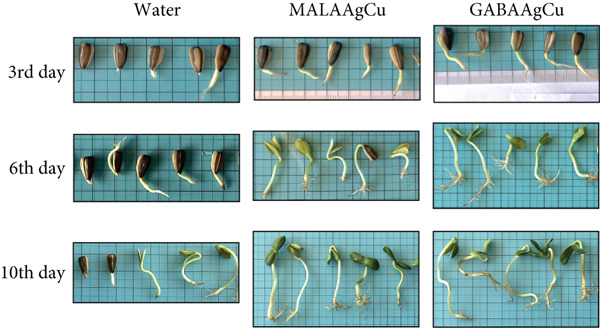
A visual comparison of seed vigor. Representative images of germinated sunflower seeds from each treatment group (water, MALAAgCu, and GABAAgCu) at 3, 6, and 10 days postpriming (Experiment 1).

Figure 8 depicts representative seedlings from each study group on Days 3, 6, and 10 postpriming. All seedlings exhibited healthy growth without atrophied hypocotyls.

#### 3.3.5. Oxidative Stress Parameters

Figures 9a, 9b, 9c, and 9d illustrate the superoxide dismutase (SOD) and GSH levels in seedlings 6 and 10 days postpriming. Compared to water‐primed seeds, nanoprimed seeds exhibited significant increases in both SOD and GSH levels. SOD levels were elevated from ~1.3‐fold (GABAAgCu) to ~4.8‐fold (MALAAgCu) on Day 6 (Figure 9a). Similarly, GSH levels increased from ~1.6‐fold (GABAAgCu) to ~5.4‐fold (MALAAgCu) on Day 6 (Figure 9b).

Figure 9T‐SOD levels: (a) 6th day, (c) 10th day. GSH levels: (b) 6th day, (d) 10th day.

**Figure:**
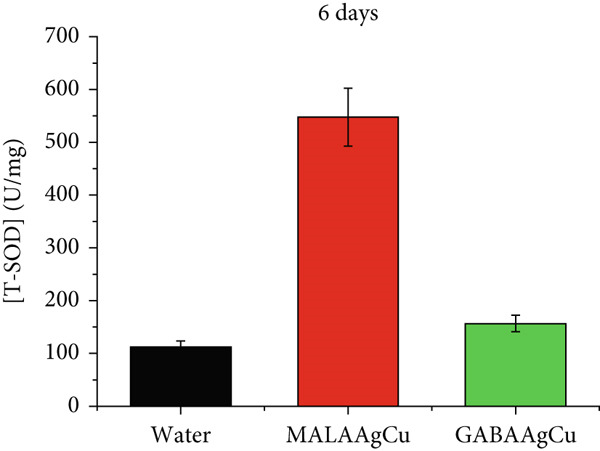
(a)

**Figure:**
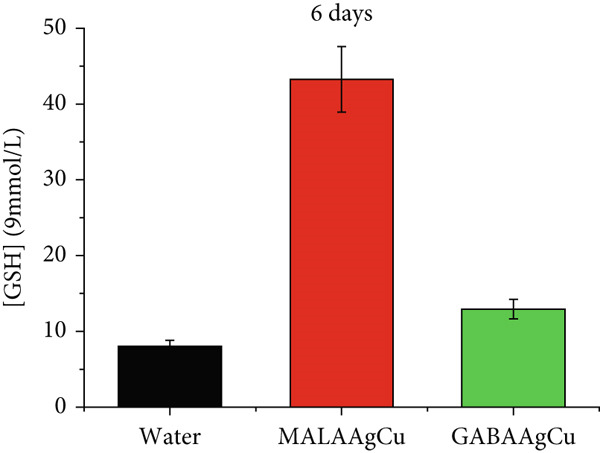
(b)

**Figure:**
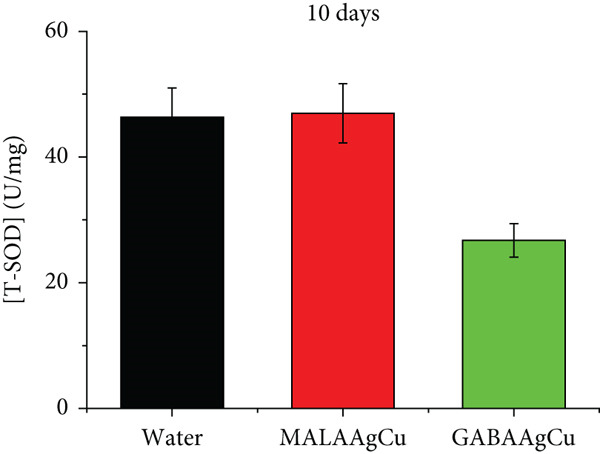
(c)

**Figure:**
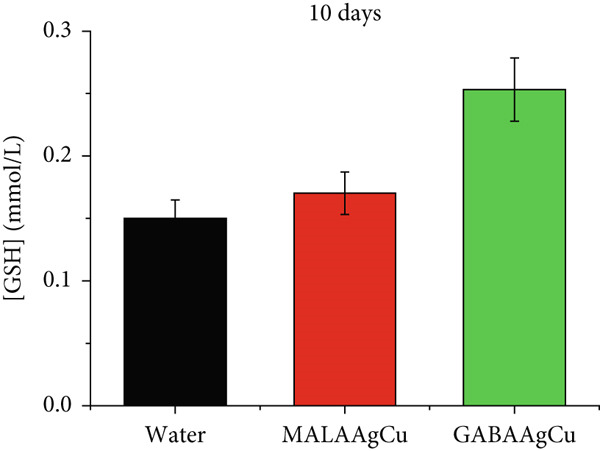
(d)

After 10 days, as shown in Figure 9c, SOD levels in MALAAgCu‐primed seeds stabilized, compared to the control, and decreased in GABAAgCu‐primed seeds. This suggests that seeds exposed to NPs may have developed alternative mechanisms to counteract oxidative stress. Despite these changes in SOD levels, GSH levels remained elevated in NP‐treated groups compared to the control (Figure 9d). This indicates that the NPs may have induced long‐lasting changes in the antioxidant defense mechanisms of the seeds.

#### 3.3.6. Chlorophyll

Figure 10a displays the average chlorophyll emission spectra of six replicate seedlings treated with water, MALAAgCu, and GABAAgCu NPs. These spectra were obtained by directly exciting the first leaves (Experiment 2, 12 days) at 435 nm. The similarity in emission profiles and intensities between NP‐treated seeds and the control suggests that the chlorophyll structure, including the ratio of Chlorophyll a, Chlorophyll b, and accessory pigments, remained unaffected.

Figure 10(a) The averaged chlorophyll emission spectra (*n* = 8) obtained under excitation at 435 nm directly over the leaves. (b) Chlorophyll content in leaves measured 12 days after priming.

**Figure:**
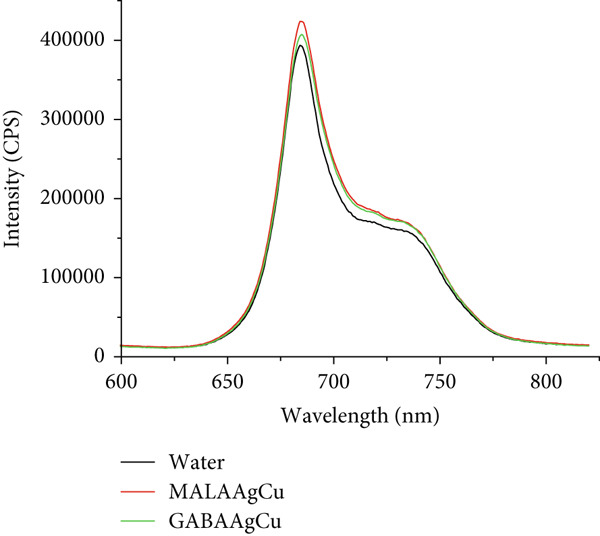
(a)

**Figure:**
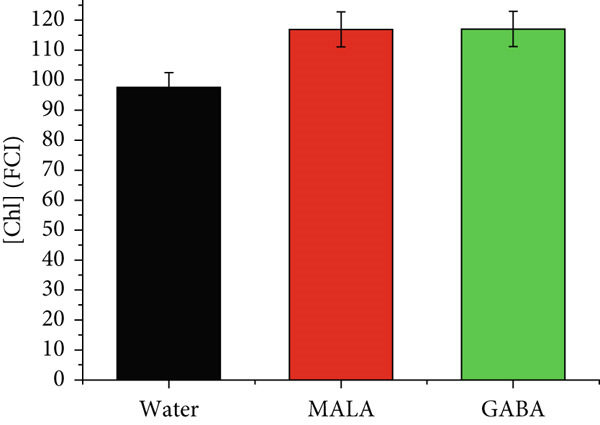
(b)

Figure 10b illustrates the chlorophyll content of seedlings treated with water, MALAAgCu, and GABAAgCu. Compared to the control group, a significant increase of ~15% (*p* < 0.05) in chlorophyll content was observed in seedlings primed with MALAAgCu and GABAAgCu.

#### 3.3.7. FLIM

**Figure 11:**
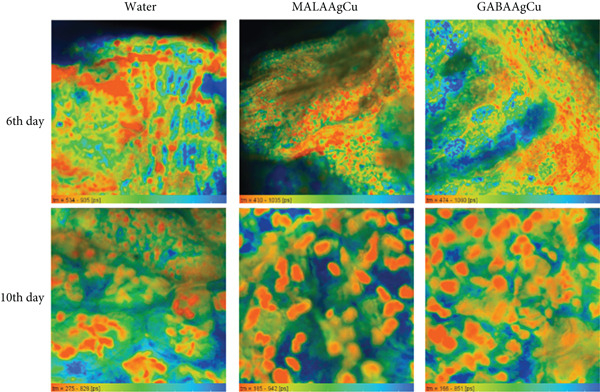
FLIM images obtained with excitation at 473 nm in the cotyledons of seeding after treatment with water, MALAAgCu, and GABAAgCu in the 6th and 10th days after priming.

Figure 11 presents FLIM images of cotyledons from seeds treated with water and NPs, acquired using 473 nm excitation [38]. Chlorophyll fluorescence within chloroplasts is visualized as orange “balls” in the images. Green and yellow fluorescence detected in the intercellular spaces is attributed to other plant fluorophores, likely flavins [39]. These flavins play crucial roles in various biological processes, especially redox reactions.

**Figure 12:**
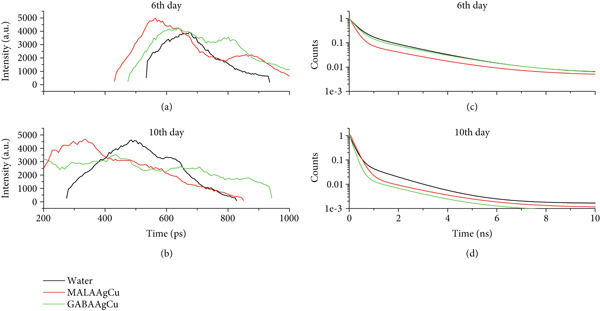
Maps and distributions of fluorescence lifetime for (a) the 6th day and (b) the 10th day. Fluorescence decay times for (c) the 6th day and (d) the 10th day.

Figure 12a,b shows the distribution of the decay time amplitude for samples on the 6th and 10th days, respectively. Figure 12a indicates peaks around 600 ps on the 6th day. On the 10th day, as shown in Figure 12b, the distribution maximum shifts to approximately 400 ps for GABAAgCu and 300 ps for MALAAgCu.

Figure 12c,d illustrates the decay time for the treatments on the 6th and 10th days, respectively. Table 1 summarizes the average lifetimes (*τ*_m_) calculated from these curves.

**Table 1:** Average lifetime (*τ*_m_) calculated using the intensity fractions *a*_1_ and *a*_2_, the pre‐exponential parameters associated with the shorter (*τ*_1_) and longer (*τ*_2_) lifetime components of a biexponential fluorescence decay model (Figure 12c,d and Equation (2)).

	**τ**_**m**_ **(6th day) (ns)**	**τ**_**m**_ **(10th day) (ns)**
Water	1.322 ± 0.003	0.652 ± 0.027
MALAAgCu	1.125 ± 0.024	0.227 ± 0.002
GABAAgCu	1.354 ± 0.004	0.458 ± 0.100

## 4. Discussions

The hybrid MALAAgCu and GABAAgCu NPs synthesis employed an optimized photoreduction protocol to facilitate precise control over NP morphology, yielding monodisperse systems critical for agricultural applications [25]. The mechanism proceeds through sequential phases: amino acid photoactivation, metallic ion reduction, nanocrystal growth, and surface stabilization. This controlled progression enables precise nanoscale dimensions and morphology engineering, which is particularly valuable for biotechnological applications [24].

The synthesized GABAAgCu (22 nm) and MALAAgCu (32 nm) NPs displayed spherical morphology with zeta potentials of −23.2 and −14.3 mV, respectively, indicating colloidal stability through electrostatic repulsion. XRD patterns (Figure 3) confirmed crystalline structures with narrow peak widths and the absence of amorphous phases, demonstrating successful amino acid capping without disrupting metal lattice formation.

Antimicrobial assessments revealed superior efficacy of bimetallic formulations: GABAAgCu achieved 96% inhibition of *S. aureus* with complete eradication of *E. coli* and *C. albicans*, while MALAAgCu showed 84% suppression of *S. aureus* and 100% elimination of other pathogens. Monometallic GABAAg and GABACu displayed limited activity (< 35% inhibition).

The NPs operate through dual antimicrobial mechanisms: (1) catalytic generation of ROS causing peroxidative membrane damage and (2) controlled release of bioactive Ag^+^/Cu^2+^ ions that inactivate microbial enzymes through thiol binding and DNA intercalation. This multifunctional action addresses critical agricultural challenges by eliminating seed‐borne pathogens without chemical residues, enhancing germination in aged seed stocks, circumventing microbial resistance development, and reducing dependence on conventional agrochemicals. The technology demonstrates promise for climate‐smart agriculture by improving seed storage stability and reducing postharvest losses.

Further enhancing these effects, antimicrobial photodynamic therapy (APDT) or PDI offers a light‐activated strategy with broad‐spectrum activity against clinically and agriculturally relevant pathogens, including resistant fungal strains [40]. PDI combines a light source of specific wavelength, such as sunlight, a photosensitizer (PS), in our case, Protoporphyrin IX metabolized by the microorganisms incubated with MALAAgCu [41] and GABAAgCu [42, 43], and oxygen to selectively kill microorganisms. The PS accumulates in microbial cells and, upon light activation, generates ROS that cause oxidative damage, leading to cell death.

Recent studies demonstrate that sunlight‐activated PDI can effectively target phytopathogenic fungi, providing a sustainable and cost‐effective approach for agricultural applications. MALAAgCu and GABAAgCu NPs exhibit strong potential as novel PS agents in this context.

The advantages of using sunlight‐driven PDI include low cost and sustainability (utilizing natural light), reduced risk of resistance (unlike conventional fungicides), and broad‐spectrum activity (effective against fungi, bacteria, and biofilms).

The observed changes in UV–Vis and FTIR spectra of posttreatment nanopriming solutions likely result from two concurrent processes: (1) leaching of seed‐derived biomolecules (phenolic compounds, flavonoids, and chromophores) during hydration and (2) active uptake of NPs into embryonic tissues.

NP interactions with seed coats can lead to increased pore size and the formation of cracks, facilitating water penetration and promoting seed hydration [10, 44].

MALAAgCu NPs induced larger pores (Figure 6), which can be due to the generation of relatively higher quantities of ROS [45]. Singlet oxygen (^1^O_2_), superoxide (O_2_^-^), hydroxyl radical (·OH), and H_2_O_2_ can induce cellular damage and structural modifications [46]. The combined effects of biomolecule release and NP internalization create a synergistic priming effect that enhances metabolic reactivation in quiescent seeds.

Nanopriming significantly enhanced germination rates compared to hydroprimed controls, with GABAAgCu‐treated seeds achieving 96.7% germination versus 80% for MALAAgCu and 66.7% for water‐treated seeds. The lower GP and Vi observed in MALAAgCu‐treated seeds compared to GABAAgCu can be attributed to oxidative stress induced by excessive ROS production, which hinders germination. The radicals generated during oxidative stress can damage the embryo, preventing proper development and emergence from the seed coat.

Several studies have highlighted the vital role of ROS in both metabolically active and quiescent dry seeds during dormancy and germination. Bailly et al. proposed the “oxidative window for germination” [47]. This window suggests that germination only occurs when the level of ROS falls within a specific range, defined by a lower and higher threshold. ROS signaling at nontoxic levels can promote positive cellular responses, including transcriptional changes and reprogramming, which can help prepare plants to adapt to abiotic stress conditions [46, 48]. Therefore, regulating ROS at these nontoxic levels could be critical in plant acclimation strategies [46].

The enzymatic antioxidant system is composed of two key enzymes: SOD and GSH reductase. SOD serves as the primary defense against ROS‐induced oxidative stress in plant cells [49]. Variations in SOD gene expression and activity reflect the extent of ROS production and oxidative stress. GSH protects plant cells from oxidative damage caused by stress [50]. These enzymes are essential for plant development and growth throughout the life cycle, even under normal conditions, due to their crucial role in fine‐tuning H_2_O_2_ metabolism and maintaining redox homeostasis. The results indicate that treatment of sunflower seeds with MALAAgCu and GABAAgCu NPs significantly enhanced their stress resistance by activating their endogenous defense system.

Plants with greater vigor tend to have elevated levels of chlorophyll and carotenoids, pigments that play a critical role in photosynthesis. Compared to the control group, a significant increase of ~15% (*p* < 0.05) in chlorophyll content was observed in seedlings primed with MALAAgCu and GABAAgCu.

The water control showed an average chlorophyll fluorescence lifetime (*τ*_m_) of 1.322 ± 0.003 ns. MALAAgCu treatment exhibited a significantly shorter lifetime (1.125 ± 0.024 ns), indicating enhanced energy transfer efficiency [51]. GABAAgCu displayed a slightly longer lifetime (1.354 ± 0.004 ns) than the control, suggesting that GABA and the protective matrix effects modify energy dissipation pathways. All systems showed reduced lifetimes by the 10th day, indicating progressive adaptation to oxidative stress and optimization of the photosynthetic apparatus. MALAAgCu achieved the shortest lifetime (0.227 ± 0.002 ns), correlated with complete recovery from the initial stress, and the highest photosynthetic efficiency among the treatments. GABAAgCu maintained intermediate quenching (0.458 ± 0.100 ns), consistent with sustained moderate stress response and balanced ROS regulation [52].

The lifetime trends support a biphasic NP action mechanism. Early phase (0–6 days): ROS‐induced pore formation enhances water uptake, while NP dissociation releases metal ions (Ag^+^/Cu^2+^) for oxidative priming and organic components (MALA/GABA) for metabolic regulation. Late phase (6–10 days): Cellular antioxidant systems normalize ROS levels, and released biomolecules enhance chlorophyll synthesis (MALA), antioxidant metabolism (GABA), and osmolyte accumulation. While MALAAgCu initially exhibited toxicity, retarding plant growth, FLIM analysis revealed a complete recovery and enhanced photosynthesis by the 10th day. The release of MALA from dissociated NPs promoted chlorophyll synthesis, contributing to the high chlorophyll levels observed in Experiment 2 plants treated with MALAAgCu, despite their lower initial vigor than GABAAgCu.

GABAAgCu‐induced oxidative stress was optimal for seed germination, stimulating beneficial cellular responses that enhanced Vi and photosynthetic performance. The gradual release of GABA within the seeds further boosted antioxidant metabolism, reinforcing stress resilience. Previous studies support these findings, demonstrating that exogenous GABA application increases soluble sugar content and reduces cellular osmotic potential in plants under diverse abiotic stresses, including flooding, drought, and salinity [16].

Future research should focus on further exploring the mechanisms of action of these NPs, optimizing their application for specific crops and environmental conditions, and assessing their long‐term impact on agricultural systems.

## 5. Conclusions

This study developed an eco‐friendly photoreduction method to synthesize amino acid–stabilized AgCuNPs, demonstrating remarkable agricultural potential. GABAAgCu NPs showed exceptional nanopriming efficacy, increasing the seedling Vi by 133%, while MALAAgCu NPs achieved a 50% enhancement. Although MALAAgCu initially induced mild phytotoxicity, it also demonstrated the capacity to improve photosynthetic performance, suggesting that concentration optimization could maximize its benefits. Collectively, these findings highlight the potential of amino acid–stabilized AgCuNPs as a sustainable nanopriming strategy to enhance crop resilience against abiotic stresses. The demonstrated antimicrobial efficacy of AgCuNPs suggests their potential applicability in mitigating biotic stresses, alongside their established role in abiotic stress resilience. The NPs demonstrated antimicrobial activity via ROS generation and sustained metal ion release, achieving 100% eradication of *C. albicans*. These integrated platforms combine abiotic stress resilience (via vigor enhancement and photosynthetic improvement) with biotic stress protection (through antimicrobial/PDI mechanisms) in a sustainable, resistance‐proof system that outperforms conventional agrochemicals, with future research needed to optimize field concentrations and develop practical delivery systems for agricultural implementation.

## Ethics Statement

The authors have nothing to report.

## Conflicts of Interest

The authors declare no conflicts of interest.

## Author Contributions

I.S.L. contributed to most parts of the article, T.D. contributed to the absorption measurements, L.T. contributed to the FLIM measurements, R.D. contributed to the SEM measurements, L.J. contributed to the XRD measurements, S.J. contributed to the supervision of the work, B.N.O.M., S.S.B., and M.R.F. contributed to determining antimicrobial activity, C.C. contributed to the writing of the article, and L.C.C. wrote the article and supervised the work.

## Funding

This work was funded by the Fundação de Amparo à Pesquisa do Estado de São Paulo (10.13039/501100001807, 2022/14030‐8).

## Data Availability

The data that support the findings of this study are available from the corresponding author upon reasonable request.
